# Laparoscopic cholecystectomy: post-operative bile and chyle leaks. A case report

**DOI:** 10.1093/jscr/rjad532

**Published:** 2023-09-24

**Authors:** Dwight Philip, Maxine Garcia, Maisha Anika, Azalia Avila, Christopher Seaver

**Affiliations:** Herbert Wertheim College of Medicine, Florida International University, Miami 33199, United States; Adult General Surgery, Memorial Hospital West, Pembroke Pines 33028, United States; Herbert Wertheim College of Medicine, Florida International University, Miami 33199, United States; Adult General Surgery, Memorial Hospital West, Pembroke Pines 33028, United States; Adult General Surgery, Memorial Hospital West, Pembroke Pines 33028, United States

**Keywords:** laparoscopic cholecystectomy, bile leak, chyle leak, case report

## Abstract

One week after an elective laparoscopic cholecystectomy at an outside hospital, a 56-year-old male presented to the emergency department with right-sided abdominal pain. Computerized tomography (CT) revealed a complex fluid collection in the gallbladder fossa. The patient underwent drain placement and received broad-spectrum intravenous antibiotics. Drain output was suspicious for a chyle leak, which was confirmed by elevated fluid triglyceride levels. Magnetic resonance cholangiopancreatography (MRCP) and hepatobiliary iminodiacetic acid (HIDA) analysis showed evidence of a concurrent bile leak. After starting a low fat, high protein diet and octreotide, a common bile duct sphincterotomy with plastic stent placement was performed. The patient’s symptoms and drain output proceeded to improve. The cause of the chyle leak is unclear. However, with consideration of the patient’s concurrent bile leak, an injury to the right major lymphatic drainage pathway and adjacent bile duct is suspected.

## Introduction

Laparoscopic cholecystectomy (LC) is a minimally invasive approach for removal of a diseased gallbladder. Of approximately 300 000 cholecystectomies performed annually in the United States, ninety percent of them are performed laparoscopically [1, 2]. Common postoperative complications reported are hemorrhage, infection, and iatrogenic common bile or hepatic duct injury. An extremely rare post-LC complication is chyle leak, with only 6 reported cases in the literature [3]. This report presents one of the few reported cases of chyle leak following LC with concurrent postoperative bile leak. We discuss associated findings, treatments, and recommendations for future medical management.

## Case presentation

**Figure 1:**
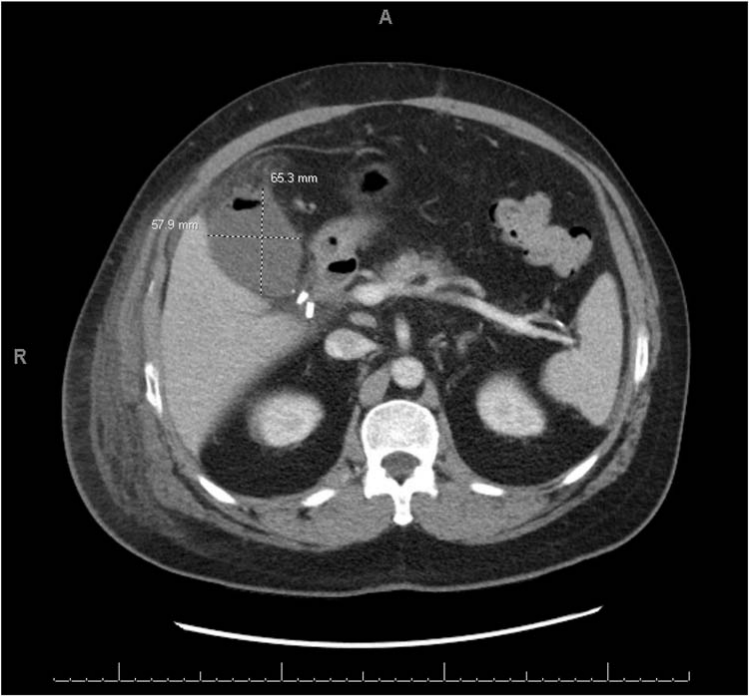
CT abdomen and pelvis with IV contrast demonstrating a complex fluid density with gaseous foci occupying the gallbladder fossa and measuring 6.5 cm × 5.8 cm × 5.5 cm.

A 56-year-old male presented with a four-day history of right upper quadrant abdominal pain. One week prior to presentation, he underwent elective laparoscopic cholecystectomy at an outside hospital where his immediate postoperative course was complicated by a penicillin allergy reaction requiring ICU level care. Upon presentation to the emergency department labs were significant for mild leukocytosis of 11.1 K/uL with neutrophilia. Lipase and liver function tests were within normal limits. CT abdomen and pelvis showed a complex fluid density and foci of gas occupying the gallbladder fossa with concern for biloma or abscess (Fig. 1).

**Figure 2:**
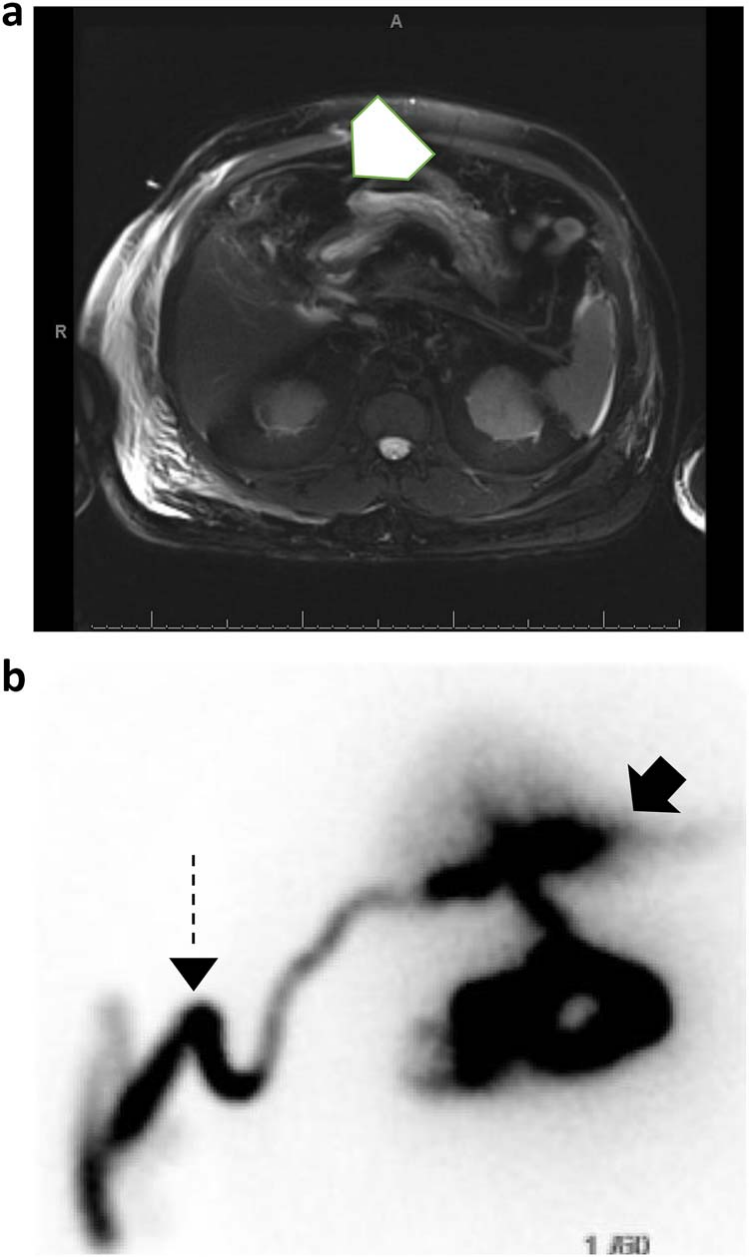
(a) MRCP with and without IV contrast identifies trace free fluid in gallbladder fossa (white arrow); (b) subsequent HIDA scan confirms right hepatic subcapsular bile leak (black arrow) adequately drained by percutaneous catheter (dashed arrow).

The patient was started on cefepime and metronidazole, and a Jackson Pratt (JP) drain was placed in the right upper quadrant by interventional radiology. The JP drain output was milky and blood-tinged with daily outputs ranging from 200 to 1500 mL. JP drain fluid triglyceride levels were > 525 mg/dL. Lymphatic injury was suspected at this time and the patient was started on a low-fat, high protein diet and octreotide. A MRCP was performed to evaluate for a possible concurrent unrecognized pancreatic injury, which showed evidence of abdominal free fluid (Fig. 2a). A subsequent hepatobiliary iminodiacetic acid (HIDA) scan revealed a bile leak that was adequately controlled by the drain (Fig. 2b). A first attempt at endoscopic retrograde cholangiopancreatography (ERCP) was unsuccessful due to ampullary stenosis. Therefore, the patient underwent advanced ERCP with endoscopic ultrasound which revealed a normal biliary tree with no obvious bile leak, at which time a common bile duct sphincterotomy with placement of a 10 French 7 cm plastic stent.

Following this procedure, the patient began having decreased output from the JP drain with noted subjective improvements in pain and progress in his diet. The patient was discharged home on hospital day 17 with JP drain care instructions, a low-fat diet, and octreotide. One month following discharge, the patient had complete resolution of symptoms with minimal drain output. A repeat ERCP showed no evidence of a persistent bile leak, and the percutaneous drain was removed.

## Discussion

Postoperative bile leak after LC is a complication with well-defined expectant management. The rate of post-LC bile leaks ranges from 1–2% [4, 5] with management typically involving drain placement, bile diversion using interventional radiology techniques, and/or rarely operative intervention [6]. In comparison, chyle leaks following LC is an atypical complication with limited evidence for successful management postoperatively [3]. In this report, we describe our treatment of a chyle leak with concurrent bile leak in a patient who underwent elective laparoscopic cholecystectomy at an outside hospital. We found that post-LC lymphatic and bile duct injuries can be successfully managed with drain placement, octreotide, low fat diet and ERCP with stent placement.

The cause of our patient’s chyle leak remains unclear. Current literature states chyle leaks post-LC are most likely iatrogenic [7]. Unfortunately, since the cholecystectomy was performed at an outside hospital, we are unsure of what occurred during the procedure or what the patient’s anatomy was at the time of procedure. Possible mechanisms of a chyle leak include the inherent anatomy of the biliary lymphatic pathways, mechanical compression of lymphatics by an inflamed pancreas, and anatomical lymphatic variants [3, 8, 9]. Considering the patient presented with a simultaneous bile leak, an injury to a lymphatic pathway adjacent to the common bile duct is likely. Damage to the right major lymphatic drainage pathway is suspected given that it anatomically descends along the cystic and common bile ducts [10]. Consequently, an iatrogenic injury to this anatomical region would result in concurrent chyle and bile leaks postoperatively.

When a patient presents with a chyle leak post-operatively, whether from extensive oncologic resections or laparoscopic cholecystectomies, it should be addressed immediately to prevent poor outcomes. High-volume chyle leaks, while rare, have been reported to have high mortality rates up to 70% [11]. Goals for therapy in the literature aim at reducing overall lymphatic flow and replacing lost nutrients. This is achieved primarily through diet modulation and pharmacotherapy. The two main therapeutic diet recommendations are Total Parenteral Nutrition (TPN) and a low-fat, high-protein diet with supplementation of medium chain triglycerides (MCTs). Addition of octreotide to either of these diet regimens led to the resolution of chyle leaks in 100% of patients [12]. Further, treatment with octreotide and TPN is shown to decrease the length of time to drain removal when compared to TPN alone [13].

Guidelines for management of chyle leak post LC are currently non-existent in the literature due to the rarity of this complication. Consensus from prior literature recommends initial conservative management and lymphangiography. Surgical consideration should occur only in patients with refractory high-volume daily outputs (>500 ml/day) or nutritional compromise [14, 15]. Out of the six total reported cases, two required surgical re-exploration. In both cases, the chyle leak was identified laparoscopically and closed with a figure-of-eight suture and fibrin glue placement [7, 14]. One patient required prolonged conservative medical management with TPN, medium-chain triglyceride supplementation and octreotide post-operatively until the drain output gradually diminished to zero after 7 months of treatment [14]. The remaining four cases were treated non-operatively with a fat-free or low-fat diet administered via either oral or parenteral nutrition which led to a gradual improvement in drainage [3, 5, 8, 16]. In this report, we presented the 7th reported case of a postoperative chyle leak following a laparoscopic cholecystectomy. While the patient underwent an ERCP for a concurrent bile leak, we were able to successfully manage his chyle leak with a low-fat, high-protein diet and octreotide pharmacotherapy with no need for further procedural intervention. Here report the successful management of the first case of a chyle leak and concurrent bile leak following elective laparoscopic cholecystectomy.
